# Beyond scale-free networks: integrating multilayer social networks with molecular clusters in the local spread of COVID-19

**DOI:** 10.1038/s41598-023-49109-x

**Published:** 2023-12-09

**Authors:** Kayo Fujimoto, Jacky Kuo, Guppy Stott, Ryan Lewis, Hei Kit Chan, Leke Lyu, Gabriella Veytsel, Michelle Carr, Tristan Broussard, Kirstin Short, Pamela Brown, Roger Sealy, Armand Brown, Justin Bahl

**Affiliations:** 1 School of Public Health, University of Texas Health Science Center at Houston, 7000 Fannin Street, UCT 2514, Houston, TX 77030 USA; 2 Institute of Bioinformatics, University of Georgia, 501 D.W. Brooks Drive, Athens, GA 30602 USA; 3 City of Houston Health Department, Houston, TX USA

**Keywords:** Diseases, Infectious diseases

## Abstract

This study evaluates the scale-free network assumption commonly used in COVID-19 epidemiology, using empirical social network data from SARS-CoV-2 Delta variant molecular local clusters in Houston, Texas. We constructed genome-informed social networks from contact and co-residence data, tested them for scale-free power-law distributions that imply highly connected hubs, and compared them to alternative models (exponential, log-normal, power-law with exponential cutoff, and Weibull) that suggest more evenly distributed network connections. Although the power-law model failed the goodness of fit test, after incorporating social network ties, the power-law model was at least as good as, if not better than, the alternatives, implying the presence of both hub and non-hub mechanisms in local SARS-CoV-2 transmission. These findings enhance our understanding of the complex social interactions that drive SARS-CoV-2 transmission, thereby informing more effective public health interventions.

## Introduction

Network models have been widely used as a valuable method to represent the complex system of human interactions underlying SARS-CoV-2 transmission^1–15^. One of the most prominent models is the scale-free network model introduced by Barabási and Albert. It is characterized by a power-law distribution of network connectivity, signifying the existence of highly connected nodes^16,17^. In tandem with this, the widely recognized small-world network model by Watts and Strogatz^18,19^ underscores the coexistence of high local clustering and short global separation. The short global separation refers to the close proximity of each individual to all other individuals, thus indicating a potential for rapid viral spread throughout the network. A scale-free model can be used in conjunction with a small-world model^20–23^. When these network models are incorporated into mathematical epidemic frameworks such as Susceptible, Infected, Recovered (SIR)^24^, Susceptible, Exposed, Infected, Recovered (SEIR)^25^, and Adaptive SIR^26^, they have capabilities of representing the propagation of pathogens more realistically. This integration results in more accurate predictions of viral transmission^27^.

Scale-free networks are characterized by their power-law distribution of network connectivity^16,17^. These networks represent a heterogeneous connectivity distribution where a few highly connected “hubs” coexist with a large number of less connected individuals. These hubs primarily contribute to the scale-free property of degree heterogeneity, which influences the speed at which a virus can spread through a contact network^27^. In the specific context of SARS-CoV-2, scale-free networks facilitate local clustering, allowing the virus to spread rapidly to many closely connected neighbors, resulting in local epidemics. This phenomenon is represented by the presence of highly connected hubs, a characteristic exhibited by the long tail of the power-law degree distribution. This long tail illustrates a unique pattern within power-law distributions in which the majority of individuals have limited connections, while a minority (referred to as the “long tail”) have extensive connectivity, thus forming hubs. These hubs promote rapid virus transmission, which is particularly evident in assortative networks^28,29^ where hubs tend to connect with other hubs, allowing the virus to spread through a large number of contacts.

In addition, a small number of highly connected infectious agents, or superspreaders, are known for their ability to infect a large number of secondary contacts. This aligns with the 80/20 Pareto principle^30^, which estimates that 20% of infectious agents cause 80% of local transmission^6^. The power-law scaling observed in the connectivity distribution of confirmed COVID-19 cases provides additional evidence for the existence of superspreaders^2^. The importance of these hubs in heterogeneous networks becomes evident in the rapid transmission of SARS-CoV-2^7,27^, where they play a key role in facilitating superspreading events and influencing the spread of the virus.

The universality of scale-free networks has been widely observed in various real-world systems, including information, biological, and technological network systems^16,17^. However, contrary to conventional belief, many of these networks do not consistently exhibit scale-free structures with power law degree distributions^31^. Instead, they display structural diversity, displaying that alternative distributions (e.g., log-normal, exponential, cutoff power laws, Weibull, etc.) may be more appropriate.

While much of the existing COVID-19 research on network-based epidemic models assumes a well-established scale-free network structure, often this is done without empirical validation specific to modeling the spread of SARS-CoV-2. Many studies rely on simulated network structures^8,32,33^, which typically have not been rigorously evaluated based on empirical network data and molecular clustering information. Consequently, it remains uncertain whether the scale-free network assumption is accurate, and thus whether scale-free models are adequate to represent the complex social interactions underlying the spread of SARS-CoV-2. This highlights the importance of considering a diverse array of network structures to better understand and improve the accuracy of modeling social networks in the context of COVID-19.

In this study, we evaluate the applicability of the scale-free network assumption for understanding the real-world complex social networks underlying the spread of SARS-CoV-2. We integrate multiple sources of real-world network data, including the SARS-CoV-2 Delta variant viral similarity sequence, personal contacts, and household co-residence. This integration provides insights into the complex social interactions that drive transmission of the Delta variant. To construct this multifaceted social network, we first infer epidemiologically linked individuals to generate putative SARS-CoV-2 transmission clusters based on the patristic distances (measured tip-to-tip distance along branch lengths of a phylogenetic tree). These phylogenetic clusters are then superimposed on distinct but overlapping, layers of social networks, using granular empirical data on personal contact and intrahousehold co-residence. This process results in the development of a multilayer social network informed by phylogeny. We then characterize the network connectivity (degree distribution) of both molecular and social networks, statistically comparing the power-law degree distribution with alternative non-scale-free distributions.

With this understanding, there is potential to refine existing public health interventions. By better understanding these networks, we can strategically prioritize effective public health responses—including vaccine distribution to communities or regions with high transmission rates, or containment efforts such as contact tracing and social distancing at highly connected hubs to suppress local transmission.

## Methods

### Study setting and data collection

The data used to estimate the SARS-CoV-2 transmission network were collected by the Houston Health Department (HHD) from individuals residing in the Greater Houston area. These data were obtained from HHD’s COVID-19 contact tracing program and HHD’s electronic laboratory reporting (ELR) system. From the ELR, we curated patient address information and COVID-19 PCR test results. All data were limited to the dates of March 1, 2020, and December 31, 2021.

Data for epidemiologic purposes were collected on positive cases using multiple methods. In the early stages of the COVID-19 pandemic, positive individuals were followed up by in-house surveillance investigators using traditional epidemiologic surveillance methods (i.e., direct interviews of case contacts and medical record abstraction) to learn more about a patient’s status and contacts. Alternatively, when the capacity for manual outreach was exceeded, the HHD worked with a contractor to automate the process. Qualtrics and telephone surveys were also used to gather additional information.

To collect contact tracing network data, for asymptomatic cases, contacts were defined as individuals exposed 24–48 h prior to an index’s positive laboratory test result; for symptomatic cases, contacts were defined as individuals exposed 24–48 h prior to the earliest symptom onset. Once an individual tested positive, HHD identified contacts through an interview, and a primary epidemiologist conducted contact tracing and interviews. Due to the volume of cases, the strategy was adjusted to assign a contact tracer after the initial primary epidemiologist collected contact information. Contact tracing outreach typically took 24–48 h.

### Construction of network data

#### Phylogenetic networks and analysis of patristic distance

To construct a molecular clustering of the Delta variant of SARS-CoV-2, we make use of a new Nextflow pipeline for generating a network database for phylogeny and epidemiologic surveillance, described in Stott, et al^34^. In this pipeline, patristic distances (tip-to-tip distance measured along branch lengths) are used to generate a genetic distance network from a time tree^35^. Using patristic distances, as opposed to measures of genetic distance that use sequence differences, creates a network with fewer tie scores and more biologically informed results as it leverages more of the information content found in the sequence alignment to generate phylogenetic trees^36^.

We collected 4176 full-genome sequences from the GISAID database^37^. These sequences represent the 545 isolates associated with contact tracing data as well as 3631 reference sequences taken from the North American region-specific Auspice source file from GISAID. The global distribution of these reference sequences used in tree building is presented in Fig. S1 in Supplemental Material. We used a Nextflow pipeline to construct a patristic distance network for our analysis. We aligned our sequences to the Wuhan/Hu reference strain using MAFFT v7.505^38^. We then used IQ-Tree2 v2.2.0.3 to generate a time tree under a generalized time reversible (GTR) substitution model with the Wuhan/WIV04/2019 sequence specified as an outgroup taxon to root the tree^39^. After constructing our time-scaled phylogenetic tree, a patristic distance network was generated using the R package ape^39^.

Smaller patristic distances imply that individuals are more likely to have been infected by a common source or within an overlapping time period. We then filtered out reference sequences from our dataset, leaving only the patristic distances between the 545 Houston isolates associated with contact tracing data. To construct putative SARS-CoV-2 transmission clusters that allow us to infer epidemiologically linked individuals, we used a 30-day patristic distance threshold to generate our phylogenetic network, i.e., 30 days of evolutionary time under our model separating the two isolates. Our rationale is as follows: Given the mean intrinsic generation time of the Delta variant of SARS-CoV-2, 4.7 days, this would correspond to chains of infection with at least 30/4.7 = 6.38 individuals^40^. To minimize noise and improve the quality of our analysis, we employed a heuristic approach by excluding connected graphs with fewer than seven individuals. This decision was based on the rationale that when dealing with smaller groups, it is unlikely that we have sampled a substantial portion of the infected population. Consequently, drawing meaningful inferences from such limited data may be challenging due to the lack of necessary context.

#### Person–person contact network

COVID-19 contact tracing data were collected by HHD through the contact tracing program implemented during the pandemic. HHD would identify contacts through an interview (e.g., phone calls, text messages), and a primary epidemiologist was assigned to conduct the contact tracing interview. The interview aimed to identify close contacts of the person under investigation. HHD defined “close contacts” as individuals who had been within 6 feet in proximity to the person under investigation for more than 15 min. We used contact tracing data collected from March 1, 2020, to December 31, 2021. The total number of individuals traced during these 21 months is 169,323, and they form a total of 166,465 connections/contacts. In this network, nodes represent individuals, and connections between a pair of individuals indicate that there was physical contact between them (i.e., they were exposed to each other).

#### Intrahousehold network

The primary address data from MAVEN were queried to create a dataset of deduplicated primary addresses, totaling 8,858,357 records. After performing text standardization and deduplication, the number of records was reduced to 3,553,801. Non-residential addresses were identified by the frequency with which they appeared in the standardized dataset. All addresses that appeared more than 15 times were considered non-residential and were subsequently excluded from address-matching algorithm. To be considered a match, two individuals must have identical (i.e., exactly matching) street numbers, apartment numbers, and ZIP codes. In addition, the street names being compared must have a Levenshtein distance greater than 0.75. To be considered co-residents, we also required that individuals matched to the same address be associated with the address with some overlap in time. To further eliminate non-residential addresses in the final matching result, we removed addresses that were associated with more than 10 individuals. The final address matching result had 1,707,126 matches, including 1,185,691 individuals and 433,162 unique addresses. The matching algorithm was developed in Python using the following libraries: Pandas, NumPy, RecordLinkage, and Address^41,42^. Using these data, we constructed the co-residency network based on address-matching results. In the co-residency network, nodes represent individuals and ties indicate co-residency between a pair of nodes. The ties were coded as “1” if two individuals shared the same address and during the same time period; otherwise, the ties were coded as “0” indicating no co-residency.

### Construction of a multilayer phylogeny-informed social network

We generated a three-layer phylogeny-informed social network, with each layer progressively increasing the complexity of the network configuration. The configuration of these layers is described as follows: (1) Phylogenetics only (Phylo-only): This initial layer consists only of phylogenetic clusters composed of epidemiologically linked individuals. It serves as a foundation for subsequent layers. (2) Phylo and person-to-person contact (Phylo + PP): In this second layer, we integrated person-to-person contact information, creating connections between individuals based on their direct social interactions. (3) Phylo + PP and household co-residence (Phylo + PP + HH). The final and most complex layer incorporates household co-residence data, connecting individuals through shared living environments. The inclusion criteria for nodes at each layer dictate that a node must either be part of the phylogenetic layer or have a direct connection to a node within that layer. This criterion ensures that all individuals in the network are phylogenetically informed. To construct our multilayer network, we used comprehensive data collected over a period of time from March 1, 2020, to December 31, 2021. Following this data aggregation, we located the individuals from the Phylo-only network within each layer. This identification process was performed using multiple identifiers, such as the individual’s name, date of birth, and specific specimen-related details (e.g., date of specimen collection). We then proceeded to include additional individuals based on their first-degree ties, defined as direct connections, to nodes within the phylo-only network. For example, in the Phylo + PP + HH network, we included individuals from both the phylogenetic layer and additional nodes that are directly connected to one of the phylogenetic nodes via person-to-person or shared household edges.

### Network visualization

We used the ‘igraph’ package^43^ in R to process the various network data objects and our visualizations were generated by the ‘GGally’ package^44^ in R.

### Scale-free network analysis

#### Evaluation of scale-free property: power law degree distribution

We used the method introduced by Broido and Clauset^31^ to test the scale-free hypothesis. This method employs a statistical goodness of fit test to assess the statistical plausibility of the power law model in representing the network’s degree sequence, specifically in the upper tail of the degree distribution. It then uses a likelihood ratio test to compare this model to four alternative non-power law distributions, each fitted to the same upper tail region^31^.

Scale-free networks are characterized by the right-tail degree distribution of the network connectivity. Specifically, the right-tail can be modeled by a discrete power law distribution defined as: 

$$P\left(k\right)=\frac{{k}^{-\alpha }}{\zeta \left(\alpha ,{k}_{{\text{min}}}\right)},$$

where $\zeta \left(\alpha ,{k}_{{\text{min}}}\right)$ is the Hurwitz zeta function and serves as the normalizing constant in the power-law distribution. The right-tail starting point is denoted by ${k}_{\text{min}}$, and the scaling parameter is $\alpha$. For a ‘strong scale-free’ structure, the power law model should achieve the following: (1) alternative models are not favored over the power law model, (2) the degree distribution should pass the power law goodness of fit test, (3) the estimated $\alpha$ should be in the range $2<\widehat{\alpha }<3$, and (4) the right-tail sample size should be sufficiently large. For a ‘weak scale-free’ structure, we only require that the power law model passes the goodness-of-fit test and that the right-tail sample size is sufficiently large (i.e., conditions 2 and 4). Finally, if the network only meet the first criterion, then we classify it as being ‘super-weak scale-free’. These classification criteria are described in more detail elsewhere^31^.

#### Statistical GOF test of scale-free network

The goodness of fit assessment test was performed to test the scale-free structure of our SARS-CoV-2 Delta variant transmission networks. The goodness of fit test uses Monte–Carlo simulation to determine whether the power law model can be considered an adequate fit to the degree distribution of the transmission networks. The data simulation was performed separately for the right and left tails. The data in the right-tail region were generated from the estimated power law model, while the left-tail were generated by bootstrapping from the left tail of the observed data. We fit the power law model to simulate the data and extracted the Kolmogorov–Smirnov (KS) statistic. By repeating this process several times, we approximated the distribution of the KS statistic under the assumption that the null hypothesis is true (i.e., the underlying generating mechanism of the data follows a power law model). By comparing the KS statistic from the actual data to the simulated data, we quantified how much the actual data deviated from the theoretical power law model. The *p*-value of the goodness of fit test is the proportion of the simulated KS statistic that is greater than the KS statistic from the observed data. A good fit is supported by $p>0.10$^45^.

#### Alternative models for the degree distribution

We compared the power law model to four alternative distributions. The competing alternative distributions included the (1) exponential distribution, the (2) log-normal distribution, the (3) power law with exponential cutoff distribution, and the (4) Weibull distribution following the method in Broido and Clauset^31^. The *exponential degree distribution* has a thin tail with an exponential decrease in the probability of highly-connected individuals. The power law distribution represents an opposite, i.e., the fat or heavy-tailed probability of degree where the decay at larger degrees is slower than exponential^46^. The *log-normal degree distribution* is one of the crossover distributions between the power law and exponential distributions. It is flexible in that it can capture large variability in the data through the $\sigma$ parameter. Depending on the value of $\sigma$, the shape of the log-normal may resemble that of a power law. The *power law with exponential cutoff* is another crossover distribution. It represents heterogeneous connectivity similarly to the power law degree distribution (as a special case), but the difference between these distributions is that the power law with exponential cutoff has less extreme heterogeneity in the right tail of the degree distribution, ensuring that the number of highly-connected individuals is limited. The high degree cutoff may represent a limit to the number of meaningful relationships with a large number of acquaintances that individuals can maintain in social networks, indicating the presence of phenomena in addition to scale-free networks^46^. The *Weibull distribution* includes a variety of shapes, ranging from light-tailed to heavy-tailed (depending on its parameters), and accommodates a wide range of network connectivity. Where necessary, the distributions were discretized using the cumulative distribution function. A variant of the likelihood ratio test was performed to compare the power law model and the alternative models. A model is considered better than another when the likelihood ratio test has $p<0.10$^31^. Subsequently, we systematically compared all possible model pairs, assigning ranks based on statistical significance. In case of  $p\ge 0.10$, we considered the comparison a tie. Our analysis treats all nodes equally, regardless of their layer or the presence of overlaps. The probability density functions for these distributions are presented in Table S1 in Supplemental Material.

## Results

### Network visualization

**Figure 1:**
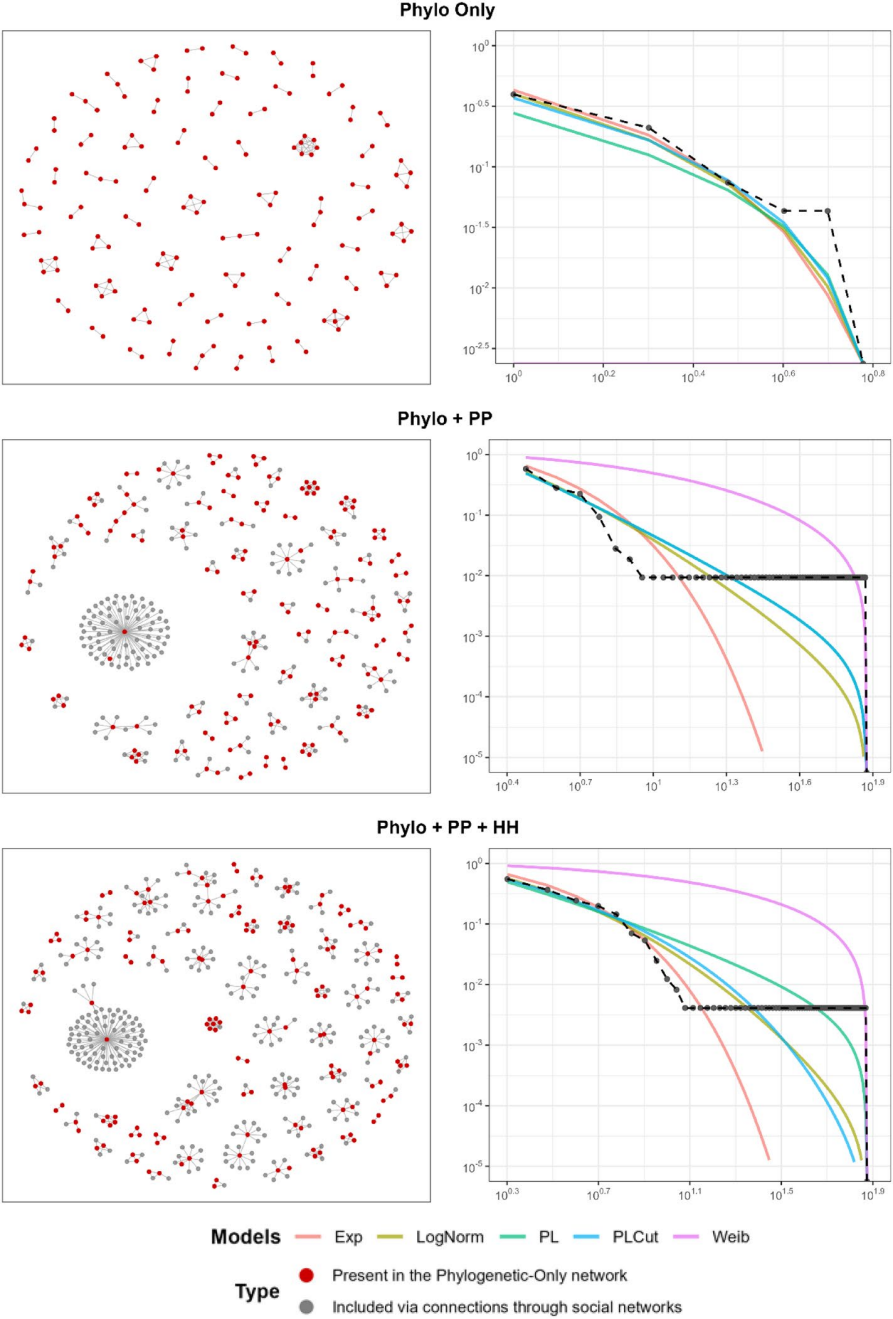
Network visualization of the multilayer COVID-19 transmission networks and the corresponding degree distributions. The networks’ sizes are 162, 352, and 464 for every added layer. Similarly, the network densities are 1.10%, 0.69%, and 0.55%. A visualization of the three networks, the empirical degree distribution (black dotted line), and the fitted models (colored solid lines). In the Phylo-only network, the power-law model was not as good as other alternative models. However, with the inclusion of social network ties (i.e., person-person contacts and intrahousehold connections), the results show that the power-law model was as good as, if not better than, the alternative models, suggesting the presence of hub and non-hub mechanisms within the SARS-CoV-2 transmission network. Optimization issues were encountered in the Weibull model of the Phylo-only network that prevented the algorithm from converging properly, which aligns with the estimated parameter $\lambda$, as it is very large $\left(\frac{1}{\lambda }=0.002\right)$. Proper convergence in models is typically indicated by a trend that aligns with the observed data. In the Phylo + PP network, the estimated PLCut model was found to be approximately the same as the PL model, causing their respective lines to largely coincide.

The left column of Fig. 1 displays visualizations of three distinct network types: Phylo-only, Phylo + PP, and Phylo + PP + HH, arranged from top to bottom.

Each network is differentiated by nodal colors, representing two primary categories: nodes present in the Phylo-only layer and nodes added through social networks (PP or HH). Table 1 provides descriptive statistics for each network, including size, density, component count, and COVID-19 positivity rate among non-Phylo nodes.

**Table 1:** Network summaries of different layers of the COVID-19 networks.

Network type	Basic network summary numbers			
	# of nodes	# of edges (density in %)	# of components	Positivity rate (%) in non-Phylo nodes
(L1) Phylo only	162	143 (1.10%)	64	N/A
(L2) Phylo + PP	352	424 (0.69%)	63	40.36
(L3) Phylo + PP + HH	464	588 (0.55%)	63	36.36

Network density is computed as the number of actual edges divided by the total number of possible edges. Number of components is defined as the number of disconnected parts within the network.

*Phylo* phylogenetic, *PP* person-person contact, *HH* intrahousehold co-residency.

The base layer, Phylo-only, comprises 162 nodes and 143 edges with a network density of 1.10%. Adding the PP layer increases the network to 352 nodes and 424 edges, subsequently reducing the network density to 0.69%. The COVID-19 positivity rate for the non-Phylo nodes was 40.36%. Adding the HH layer further extends the network to 464 nodes and 588 edges with a network density of 0.55% and a COVID-19 positivity rate for non-Phylo nodes of 36.36%. Regarding the overlaps in nodes and edges across these network layers within the context of the Phylo + PP + HH network—comprising a total of 464 nodes—1 node (0.22%) is shared between the Phylo and HH, 77 nodes (16.59%) overlap between the Phylo and PP, 41 nodes (8.84%) are shared between the PP and HH, and 83 nodes (17.89%) are concurrent members of the Phylo, PP, and HH. With respect to the 588 edges of the network, there are 2 edges (0.34%) that overlap between Phylo and HH, 54 edges (9.18%) between Phylo and PP, 67 edges (11.39%) between PP and HH, and 44 edges (7.48%) that span all three categories of Phylo, PP, and HH.

### Scale-free network analysis

The right column of Fig. 1 displays the empirical degree distribution (depicted by a black dotted line) juxtaposed with the fitted models (illustrated with colored solid lines). The model includes power law (green), exponential (orange), log-normal (yellow), power-law with an exponential cutoff (blue), and Weibull (pink) degree distributions. Table 2 presents results from a comprehensive scale-free network analysis, listing model estimates, the goodness of fit test, AIC, likelihood ratios test composed of alternative models, and a ranking based on each network layer’s model performance.

**Table 2:** Scale-free network analysis results along with candidate models’ performance at different network layers.

Network layer/candidate models	Scale-free analysis			
	Model estimates	AIC	Ranking by LRT	Conclusion
(L1) Phylo only				
Power Law ($\alpha$, $k$-min, GoF $p$)	(2.249, 1, < 0.01)	416.630	4	Not scale-free: Power Law GoF test failed, and some models were better than Power Law
Exponential ($\lambda$)	(0.836)	393.416	**1**	
Log-Normal ($\mu$, $\sigma$)	(0.187, 0.733)	395.535	**1**	
Power Law with Exponential Cutoff ($\lambda$, $\alpha$)	(1.000, 0.433)	395.035	**1**	
Weibull $\left(\beta , \frac{1}{\lambda }\right)$	(0.811, 0.002)	394.378	5	
(L2) Phylo + PP				
Power Law ($\alpha$, $k$-min, GoF $p$)	(3.173, 3, < 0.01)	359.328	**1**	Super-weak scale-free: Power Law GoF test failed but Power Law was better than or as good as the alternative models
Exponential ($\lambda$)	(0.434)	396.385	**1**	
Log-Normal ($\mu$, $\sigma$)	(− 5.123, 1.711)	358.240	**1**	
Power Law with Exponential Cutoff ($\lambda$, $\alpha$)	(3.173, 4.551e−15)	359.328	**1**	
Weibull $\left(\beta , \frac{1}{\lambda }\right)$	(0.615, 7.799)	369.637	5	
(L3) Phylo + PP + HH				
Power Law ($\alpha$, $k$-min, GoF $p$)	(2.436, 2, < 0.01)	893.425	**1**	Super-weak scale-free: Power Law GoF test failed but Power Law was better than or as good as the alternative models
Exponential ($\lambda$)	(0.417)	916.050	**1**	
Log-Normal ($\mu$, $\sigma$)	(− 0.027, 1.030)	879.171	**1**	
Power Law with Exponential Cutoff ($\lambda$, $\alpha$)	(1.815, 0.092)	884.232	**1**	
Weibull $\left(\beta , \frac{1}{\lambda }\right)$	(0.564, 16.116)	883.130	5	

Rankings were determined by the likelihood ratio test (LRT). In cases where there is no significant difference between the models, the models are considered tied in their ranking.

*Phylo* phylogenetic layer, *PP* person-person contact layer, *HH* intrahousehold co-residency layer.

The best performing models by LRT ranking are in bold.

In the Phylo-only network, the power law model yielded estimates of ${\widehat{k}}_{{\text{min}}}=1$ and $\widehat{\alpha }=2.25$. However, the goodness-of-fit test indicated a poor fit for the power law model. When compared to the alternative models, the exponential distribution, log-normal distribution, and power law with exponential cutoff all performed better than the power law model.

For the subsequent network layer (Phylo + PP), the power law model generated ${\widehat{k}}_{{\text{min}}}=3$ and $\widehat{\alpha }=3.17$. The goodness of fit test produced a *p*-value less than 0.01. When compared with alternative models, the power law model was better than, or at least as good as, all alternative models.

We obtained similar results when we added the intrahousehold layer (HH) of the network to the previous layer. The power law model’s estimates were ${\widehat{k}}_{{\text{min}}}=$ 2 and $\widehat{\alpha }=$ 2.44. A *p*-value less than 0.01 was also observed in the goodness of fit test. When compared to the alternative models, the power law model was also better than, or at least as good as, all the alternatives.

Overall, our network data did not provide enough evidence to support a strong scale-free network structure in our empirical SARS-CoV-2 transmission network data. The goodness-of-fit tests consistently negated the power law model across all network layers. At best, the empirical networks could be considered as ‘super-weak scale-free’, as the power law model did not fit the empirical degree distribution well.

## Discussion

Our study evaluated the applicability of a scale-free network model in representing the real-world complex social interactions underlying the spread of SARS-CoV-2. These interactions encompass multilayer molecular phylogenetic clustering, personal contacts, and intrahousehold co-residence. For the network solely based on phylogenetics (Phylo-only), our findings indicate that it is improbable to observe a scale-free power law structure, which would imply the existence of hubs influencing the spread of the Delta variant of SARS-CoV-2. Instead, other connectivity distributions, such as exponential, log-normal, and power law with an exponential cutoff, appear to be more plausible.

The first plausible connectivity pattern we identified, the exponential distribution, indicates that the phylogenetic connections between those infected by the Delta variant demonstrate a low variance in connectivity distribution. This stands in contrast to networks characterized by highly-connected hubs. Such a homogeneous network may exhibit a more evenly distributed local clustering pattern. The second observed plausible connectivity pattern, the log-normal distribution, suggests a broad range of degree distributions. Most individuals have a moderate number of connections, falling in the middle, while only a few fall outside this bracket. Similar to the exponential distribution, log-normal networks also display a local clustering pattern that is more evenly distributed. Furthermore, they align with the characteristics of a scale-free power law connectivity distribution. The third connectivity pattern, characterized by a power law with an exponential cutoff for the right-tail truncated distribution, effectively limits the number of highly-connected individuals. This results in more homogeneous clustering patterns compared to the power law without the cutoff. By introducing this cutoff, the variance in local clustering is reduced, resulting in a more consistent and homogeneous network.

Generalizing these results poses a challenge due to our limited sampling among SARS-CoV-2 positive individuals. Our selected patristic distance threshold of 30 days will lead to transmission chains of up to length 7 between connected sample pairs. These long transmission chains, combined with our limited sampling of SARS-CoV-2 samples in the area, will obfuscate much of the underlying network structure when using only the phylogenetic network. Investigating these biases in sampling and elucidating how they affect downstream analyses will be important in future phylogenetic network analysis. One potential avenue for further study is to develop more complex hierarchical Bayesian models that might allow us to incorporate models for sampling bias into our network inferences.

When a social layer of personal contact ties, irrespective of the COVID-19 infection status, is superimposed on the phylogenetic clusters (Phylo + PP), our result indicates the plausibility of a scale-free power law degree distribution. This underscores the significance of social contacts in forming hubs. These hubs consist of a few highly-connected individuals who have increased opportunities to interact with infected network members. If these individuals become virus carriers, they are highly likely to spread the virus not only within the personal network but also more globally by bridging less connected individuals (disassortative networks) or by linking different highly-connected hubs (assortative networks) whose members may carry different viral variants (from different phylogenetic clusters). This will lead to multiple disparate viral variants being present within the same social network cluster.

Alternative degree distributions such as exponential, log-normal, and power law with exponential cutoff remain plausible within the personal contact network surrounding the delta-variant SARS-CoV-2 clusters. These connectivity distributions, discussed above, demonstrate more homogeneous connectivity patterns and fewer outliers for highly-connected individuals, making them appropriate for representing the local clustering of social connections surrounding SARS-CoV-2 delta-variant clusters. These trends persist even when the social layer of intrahousehold networks (Phylo + PP + HH) is included. In all cases, whether considering hubs (plausibility of power law distribution) or non-hubs (plausibility of alternative distributions), both mechanisms can contribute to viral spread throughout the population.

Our study also has several limitations. First, our phylogenetic data focused primarily on the Delta variant. This limits the generalizability of the results to other SARS-CoV-2 variants or airborne infections with different basic reproduction numbers, which represent the average number of secondary infections caused by an infected individual. Future research should investigate multiple variants to comprehend complex social interactions and variant-specific behaviors of viral propagation. Second, the maximum sample size we used was determined by our focus on samples from the Delta sequence data. While this ensures a biologically informed dataset, it may limit the breadth of our conclusions given the perceived small network size. To address this issue, we conducted a subsequent sensitivity analysis that included all accessible social network data collected between March 1, 2020 and December 31, 2021—with sample sizes of 29,111 for the person-to-person contact network (PP) and 28,389 for the combined PP and intrahousehold co-residence (PP + HH) network—both of which networks suggested the power-law model as not a good fit (*p* < 0.001), reinforcing our observations that empirical social networks often deviate from scale-free structure. Third, missing links due to underreporting of contacts or limited contact tracing resources may have resulted in an incomplete representation of transmission patterns. Fourth, our study is limited in the use of aggregated social network data, based on our assumptions on the resilience of pre-existing close social ties during the pandemic^47^. The contact tracing data and test data we used presented challenges in ascertaining the duration of co-residence. Adequately capturing long-term change is particularly challenging given the complex interplay between social interactions and the biology of specific variants. Future research should examine network dynamics including network growth and preferential attachment^16,17^. Fifth, our study focused on the scale-free power law to analyze the structure of the SARS-CoV-2 transmission network. On the other hand, properties of small-world networks^18,19^ represent a different structure that is often used in network-based epidemic models for COVID-19 to represent contact networks^4,13–15^. These networks are characterized by their high local clustering coexisting with short global separations or shortcuts that connect distant individuals. Future research would benefit from investigating small-world properties and other network topologies, such as community modularity^48^ to gain insight into both the local and broader transmission implications of SARS-CoV-2 and to provide a solid foundation for future studies. Sixth, the phylogenetic network might be limited due to our large patristic distance threshold (causing connected pairs to represent chains of up to 7 unsampled people) and low percentage of cases sequenced. Given the high percent positivity rate, we can infer that we do not have sequence data for the vast majority of cases. Future research should investigate how sampling may affect our phylogenetic network hierarchical Bayesian modeling and investigate questions around differential sequencing availability by demographic characteristics. Finally, although beyond the scope of this study, different connectivity patterns of social interactions at common outbreak sites (e.g., schools, homeless shelters, nursing homes) may drive viral transmission. Future research could gain additional insight into the behavior of viral transmission by incorporating spatial information.

## Conclusion

In conclusion, this study addresses a knowledge gap by evaluating the applicability of widely used scale-free network models in capturing the complex social interactions underlying the phylogenetically supported SARS-CoV-2 spread. Our results suggest that although scale-free network properties have been widely adopted in COVID-19 network-based epidemic models, they may not fully capture the intricacies of real-world social interactions influencing SARS-CoV-2 spread. As interactions between individuals with similar viral strains increase, network connectivity reflects a power-law scale-free network, and multiple forms of degree distributions can represent this connectivity. This diversity in connectivity patterns highlights two points: first, the importance of targeting local clusters with interventions such as vaccination or contact tracing to disrupt transmission pathways; and second, the need for a comprehensive approach to capture the consistent clustering pattern of SARS-CoV-2 within local communities. Ultimately, understanding these complex social dynamics could better prepare us for future pandemics and improve public health responses.

### Supplementary Information

Supplementary Information.

## Data Availability

Data were collected through COVID-19 surveillance by the Houston Health Department which did not require informed consent. This study was approved by the Institutional Review by the Committee for the Protection of Human Subjects at the University of Texas Health Science Center at Houston (HSC-SPH-20-1022). The de-identified data were analyzed in accordance with a Memorandum of Understanding for data sharing between the Houston Health Department and the University of Texas Health Science Center at Houston. All methods were carried out in accordance with relevant guidelines and regulations. The data that support the findings of this study are available from the Houston Health Department, but restrictions apply to the availability of these data. Data are however available from the authors upon reasonable request with the written permission of the Houston Health Department. Permission from the Houston Health Department may be requested by contacting the Investigative Review Committee (analysisdatarequest@houstontx.gov). More: www.houstonhealth.org/about/investigative-review-committee. R programming codes for this paper are located at: https://github.com/Fujimoto-lab-UTHealth-HHD/Beyond-scale-free-networks
