# Supplementary material for: Beyond scale-free networks: integrating multilayer social networks with molecular clusters in the local spread of COVID-19
Source: Sci Rep. 2023 Dec 9;13:21861. doi: 10.1038/s41598-023-49109-x (PMC10710469; doi:10.1038/s41598-023-49109-x)
Supplement: Supplementary file 1 — Supplementary Information. [file 41598_2023_49109_MOESM1_ESM.docx]

**Supplemental Material**


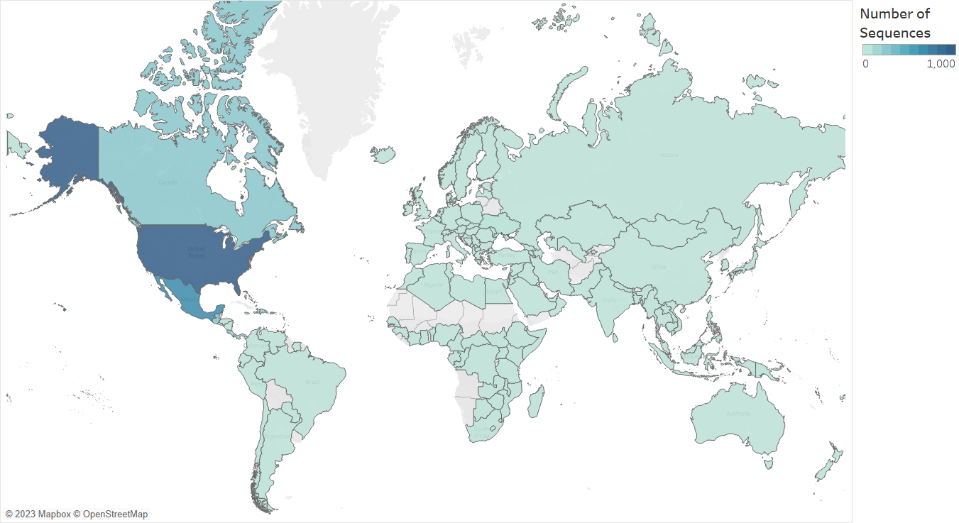


**Figure S1**: Geographical distribution of SARS-CoV-2 reference sequences

**Legend**: This visualization illustrates the global distribution of our dataset excluding 545 Houston samples, which contains a total of 3,631 reference sequences. North America contributed the majority, with 2,925 sequences, followed by Europe (261 sequences), Asia (182 sequences), Africa (110 sequences), Oceania (81 sequences), and South America (72 sequences). The map uses gradient shading, with darker colors indicating regions with a higher concentration of sequences. While North America has the highest concentration of sequences, several other regions and countries contribute to our dataset. This diverse representation indicates the broad geographic context of our study of the SARS-CoV-2 transmission network.

**Table S1:** Probability density functions of related degree distributions.

| **Distribution** | **Probability Density Function** |
| --- | --- |
| Power Law ($\alpha,k_{min}$) | $p\left( k \right)=\frac{k^{-\alpha}}{\zeta\left( \alpha,k_{min} \right)}$ |
| Exponential ($\lambda$) | $f\left( x \right)=\lambda e^{-\lambda x}$ |
| Log-Normal ($\mu$, $\sigma$) | $f\left( x \right)=\frac{1}{x\sqrt{2\pi\sigma^{2}}}e^{-\left( \ln x-\mu\right)^{2}/\left( 2\sigma^{2} \right)}$ |
| Power Law with Exponential Cutoff ($\lambda$, $\alpha$) | $f\left( x \right)=\frac{\lambda^{1-\alpha}}{\Gamma\left( 1-\alpha\right)}x^{-\alpha}e^{-\lambda x}$ |
| Weib $\left( \beta, \frac{1}{\lambda} \right)$ | $f\left( x \right)=\beta\lambda^{\beta}x^{\beta-1}e^{-\left( \lambda x \right)^{\beta}}$ |

Note: $\zeta\left( \alpha,k_{min} \right)$ is the Hurwitz zeta function serving as the normalizing constant in the power-law distribution.
